# Metagenome-assembled genomes reveal novel diversity and atypical sources of a superbug

**DOI:** 10.1128/spectrum.00106-25

**Published:** 2025-03-18

**Authors:** Rafael López-Sánchez, Alejandro Aguilar-Vera, Santiago Castillo-Ramírez

**Affiliations:** 1 Programa de Genómica Evolutiva, Centro de Ciencias Genómicas, Universidad Nacional Autónoma de México, Cuernavaca, Mexico; Università Roma Tre, Roma, Italy

**Keywords:** superbug, genomic epidemiology, metagenomics, *Acinetobacter baumannii*, metagenome-assembled genomes

## Abstract

The genomic epidemiology of *Acinetobacter baumannii* has been based on culture-dependent whole-genome sequencing. However, this approach neglects sources that are not amenable to microbial culture. Here, we show that metagenome-assembled genomes (MAGs) are useful in appraising the neglected diversity of *A. baumannii* from unconventional sources. We analyzed 22 MAGs in the context of genomes from the main human international clones and some animal and plant isolates. Given the known ecology of the species, some of these MAGs came from unexpected sources, such as the New York City subway or a kitchen counter. Our global phylogeny, with 240 genomes, demonstrated that although some MAGs were associated with some human international clones, most of them were not and were distantly related to the human clones. Furthermore, we found that these MAGs have antibiotic-resistance genes. Our results show that many MAGs represent novel lineages with some important resistance genes coming from unexpected sources. Our work illustrates a powerful approach to studying the neglected diversity of this superbug from uncommon sources. We anticipate that in the coming years, MAGs will be employed to analyze unconventional sources for other important superbugs.

**IMPORTANCE:**

The genomic epidemiology of *Acinetobacter baumannii* has been based on cultivated bacterial isolates. This disregards *A. baumannii* diversity from sources not amenable to microbial culture. In this study, we demonstrate that metagenome-assembled genomes (MAGs) are very helpful in assessing the understudied diversity of *A. baumannii* from atypical sources. Our results show that many MAGs represent novel lineages with important resistance genes coming from unexpected sources. We anticipate that in the coming years, approaches similar to ours will be employed to delve into different sources for other important superbugs.

## OBSERVATION

Antimicrobial drug resistance is a major public health crisis. In this respect, the ESKAPE (*Enterococcus faecium*, *Staphylococcus aureus*, *Klebsiella pneumoniae*, *Acinetobacter baumannii*, *Pseudomonas aeruginosa*, and *Enterobacter* spp.) bacteria are a major cause of human deaths due to antibiotic-resistant infections. Whole-genome sequencing (WGS) has been essential in characterizing the genetic structure and transmission patterns of these bacteria. However, given the culture dependence of standard WGS, many potential sources of these superbugs have not been explored.

In this respect, metagenome-assembled genomes (MAGs) inferred from metagenomic culture-independent data have been shown to be useful in understanding bacterial diversity from different environments that are not very amenable to culture-dependent approaches (1). As a proof of concept, we gathered high-quality MAGs of *A. baumannii* and demonstrated that they can be a helpful tool for studying atypical sources of superbugs and uncovering untapped diversity within these bacteria. According to some suggestions from the Genomic Standard Consortium (2), we included only MAGs with completeness >95% and contamination <5%, as well as those with defined geographic location. We chose *A. baumannii* as it is one of the most relevant bacteria in terms of human nosocomial infections (3), and it is even considered a One Health problem for which we need to increase our research efforts on its non-human populations (4, 5). Our data show that many MAGs represent novel lineages coming from unexpected sources.

**Fig 1:**
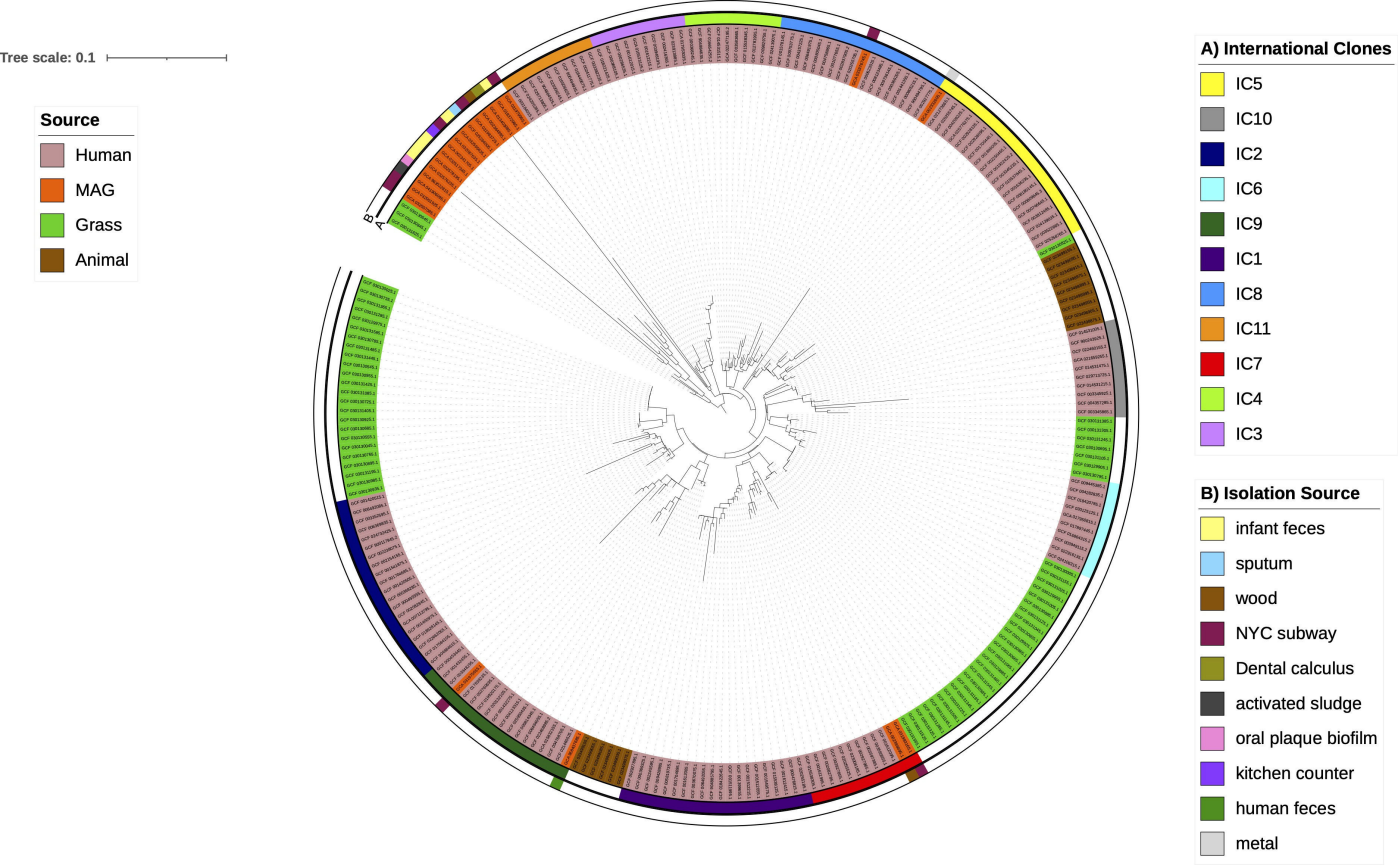
Phylogeny showing the MAGs in the context of the known diversity of *A. baumannii*. Maximum likelihood phylogeny based on the single gene families without recombination. Isolates are colored-coded, showing human, animal, and grass lineages and MAGs. The outer ring (B) provides the source of the MAGs, whereas the inner ring (A) highlights the human international clones. The scale shows the phylogenetic distance, measured in substitutions per site.

**Fig 2:**
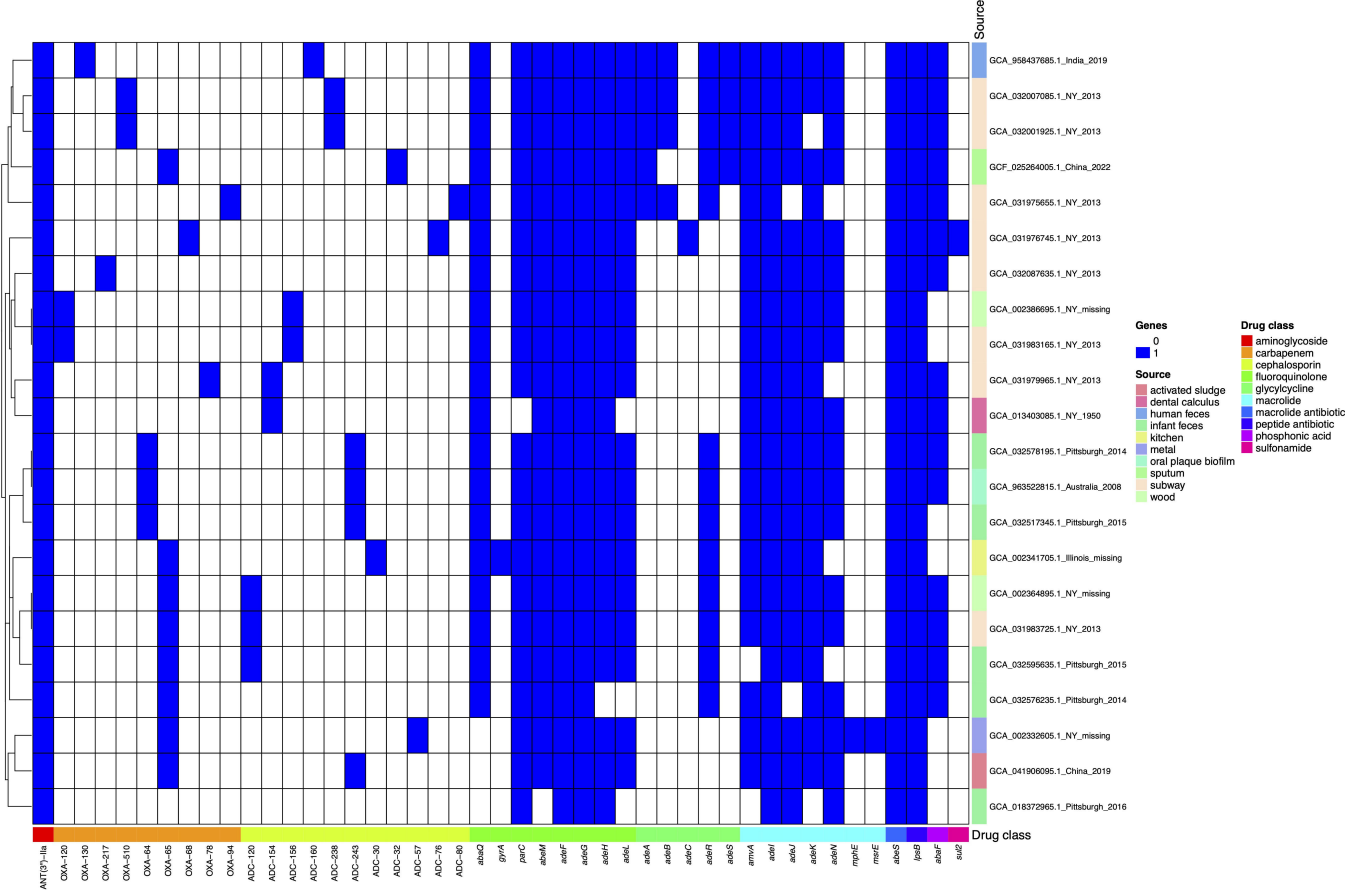
Prediction of the antibiotic resistance genes. The different antibiotic resistance genes are shown in the heat map. The *in silico* prediction was conducted using CARD. In the case of gyrA*,* blue means that the isolate has the SNP associated with the resistant phenotype. MAGs are to the right of the heat map, and the antibiotic resistance genes are at the bottom. The vertical vector to the right shows the source of the MAGs, whereas the bottom horizontal vector denotes the drug class.

To make reliable inferences, we selected only MAGs that were already available on the National Center for Biotechnology Information (NCBI) database and had sound completeness (>95%) and hardly any contamination (<5%) (see Table S1). We found 22 MAGs coming from four different countries, namely, Australia, China, India, and the USA. These MAGs were recovered from 10 distinct sources (see Table S1; Fig. 1): human feces, oral plaque biofilm, a kitchen counter, metal, wood, dental calculus, the New York City (NYC) subway, sputum, and activated sludge. Most of the MAGs were inferred from the samples isolated between 2008 and 2022, but one was from 1950, and 3 MAGs did not have information. Importantly, many of these MAGs were recovered from very atypical sources, that is, environments not previously described or hardly ever described as sources for this species. For instance, they were found in activated sludge, a kitchen counter, or the NYC subway. To put these MAGs in the context of the global *A. baumannii* population and better understand their diversity, we constructed a phylogeny following previous studies (6, 7), including genomes from the main human international clones (ICs) and some isolates recently described for animals (pigs and cattle) and grass (8, 9). Table S1 lists all the animal, ICs, and grass isolates selected (228 genomes), which were downloaded from the NCBI. A global phylogeny consisting of 250 genomes was constructed (see Methods). The phylogeny (Fig. 1) shows that most MAGs (13/22, 59%) form a diverse clade that is not closely related to any of the major human ICs or animal or plant isolates, implying that this clade has some overlooked diversity of this species. However, we also observed that few MAGs clustered within or next to major human ICs. For instance, we noted two MAGs were associated with IC7, two with IC9, one with IC8, and another with IC5 (see Fig. 1). Thus, while a few MAGs are closely related to some important human ICs, many MAGs are very distantly related to the human ICs, and we consider they represent novel diversity within species. We also determined the resistome for the MAGs conducting an *in silico* prediction by means of CARD (10) (see Methods). Figure 2 shows a heat map visualizing the resistome of the MAGs. The MAGs have important intrinsic resistance genes, such as efflux pumps and intrinsic oxacillinases (OXA-51-like family). We noted two types of efflux pumps and several allelic variants of the OXA-51-like family. These MAGs also have several ADC β-lactamase variants. Of note, compared to *A. baumannii* isolates of human clinical origin, which have a vast amount of acquired resistance genes (11), the MAGs analyzed here had hardly any acquired resistance genes. Nonetheless, these MAGS have a few acquired resistance genes of clinical relevance, such as *sul2* or *mphE* (see Fig. 2). We acknowledge that the paucity of acquired antibiotic resistance genes might also be due to the very nature of the MAGs, as in some instances, not all the actual genes for an isolate might be recovered in a MAG. Collectively, these data show that although MAGs do not have as many antibiotic resistance genes as human clinical isolates, they do have important intrinsic antibiotic resistance genes and few acquired antibiotic resistance genes.

In conclusion, these results show that many MAGs represent novel lineages with relevant resistance genes coming from unexpected sources, given the current known ecology of the pathogen. If we want to understand the global, multi-host epidemiology of *A. baumannii* (12), we should go beyond cultivated isolate genomes and also consider MAGs. However, we should ensure compliance with some minimum standards of quality and metadata (2). Of note, MAGs can be useful beyond this species and help uncover genomic diversity for other species within the genus. For instance, in the recently conducted largest genomic epidemiology study of *Acinetobacter junii* (13), we noted that several genomes included were reconstructed from metagenomes (i.e., MAGs; see Table S1 in Aguilar-Vera et al.[13]). In a broader context, our study illustrates that MAGs can be a powerful approach to studying the neglected diversity of superbugs from uncommon sources. We anticipate that in the coming years, MAGs will be employed to analyze unconventional sources for other ESKAPE pathogens.

All the genomes were downloaded from the NCBI database and are listed in Table S1. We used CheckM v.1.1.3 (14) to evaluate the completeness and contamination of all the genomes analyzed. Values of completeness and contamination, as well as metadata for all the genomes, are reported in Table S1. To ensure high-quality MAGs, only those showing values of completeness >95%, contamination <5%, and data on geographic location were selected. For consistency, all the genomes were re-annotated using PROKKA v1.12 (15). Single gene families were identified by running a pan-genome analysis via Roary (16). Individual gene alignments were constructed using FSA (17) and concatenated with an in-house PERL script. The global phylogeny was constructed on the concatenated alignment of all the single gene families without recombination, and the maximum likelihood was run on the concatenated alignment employing RAxML v8 (18). We used the GTR + I model to run the phylogeny. We also determined the resistome for the MAGs using CARD (10), considering a coverage of ≥70% and an identity percentage of ≥95%.

## Data Availability

All the genomes used in this study are listed in Table S1. This table provides the GenBank or RefSeq assembly accession numbers.

## SUPPLEMENTAL MATERIAL

Table S1 spectrum.00106-25-s0001.xls Genomes and MAGs used in this study.

ASM does not own the copyrights to Supplemental Material that may be linked to, or accessed through, an article. The authors have granted ASM a non-exclusive, world-wide license to publish the Supplemental Material files. Please contact the corresponding author directly for reuse.
